# Multiscale spatial heterogeneity of population aging in relation to its influential factors: a case study in the Shaanxi-Gansu region, China

**DOI:** 10.3389/fpubh.2025.1551287

**Published:** 2025-03-27

**Authors:** Fei Long, Qing Luo, Zirui Li

**Affiliations:** School of Mathematics and Physics, Wuhan Institute of Technology, Wuhan, China

**Keywords:** population aging, Shaanxi-Gansu region, spatial heterogeneity, influential factors, multiscale geographically weighted regression

## Abstract

**Objective:**

With the extension of life expectancy and persistently low birth rates, population aging has become a pressing issue in China. This study investigates and visualizes the multiscale spatial heterogeneity of population aging and its influential factors (demographic, socioeconomic, healthcare, and natural environmental factors) across the Shaanxi-Gansu region in northwestern China for 2010 and 2020, and aims to offer some insights for designing localized aging policies to promote an older adult-friendly society.

**Methods:**

Using county-level census data and nighttime light data, spatial autocorrelation analysis and multiscale geographically weighted regression were applied to explore spatial patterns of aging and the varying impacts of different factors across scales.

**Results:**

The results reveal progressive population aging and significant spatial heterogeneous impacts in the region. In 2010, demographic factors had global effects, economic factors had local effects, and environmental factors influenced at regional scales. By 2020, healthcare factors exerted global impacts, while the spatial influence of the other factors varied within each category.

**Conclusion:**

The Shaanxi-Gansu region experienced accelerated aging along with distinct spatial–temporal heterogeneity in aging patterns. The scale and magnitude of the impacts from four types of influencing factors also shifted over the study period. These findings highlight the importance of addressing aging challenges by considering the specific local characteristics of each area.

## 1 Introduction

**Figure 1:**
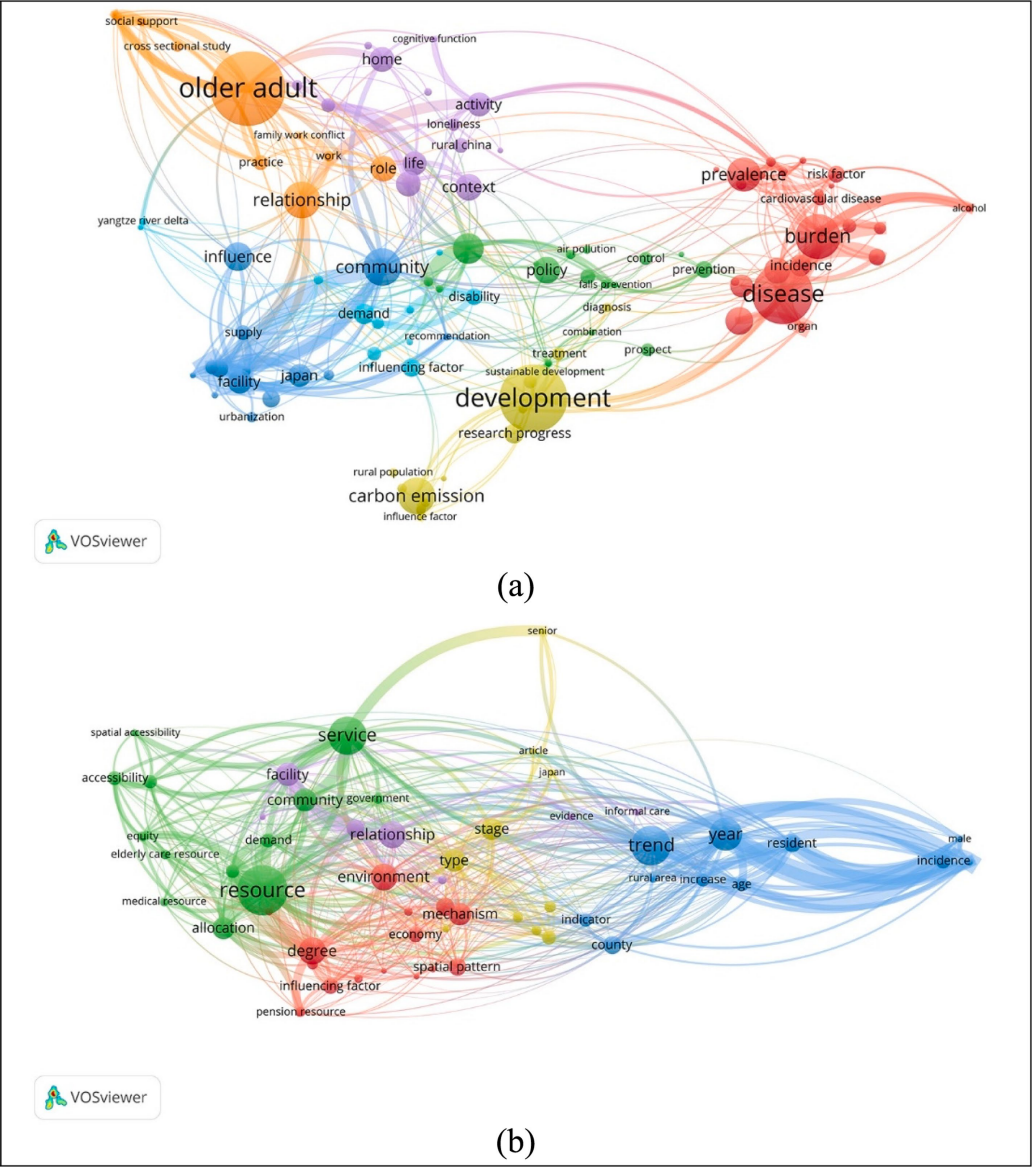
Keywords co-occurrence maps of population aging researches relating to China from 1985 to 2024 by VosViewer (76), keywords were searched in Web of Science Core Collection database and Chinese Science Citation database: **(a)** Keywords co-occurrence map of 1,309 papers searched by topics “population aging” and “China”; and **(b)** Keywords co-occurrence map of 122 papers searched by topics “population aging,” “China,” and “spatial”.

China is undergoing rapid population aging, with the median age dramatically rising from 21.4 in 1984 to 39.1 in 2024 (1), and by 2023, 15.4% of the population was aged 65 or older (2), far surpassing the international threshold of 7% that defines an aging society. This trend which is driven by increased life expectancy and persistently low birth rates, poses a significant challenge. Furthermore, the spatial distribution and underlying mechanisms of population aging differ across regions (3, 4), complicating efforts by central and local governments to address this issue effectively. Researches of China’s population aging mainly focus on three aspects (Figure 1a): disease burden and healthcare needs of the older adult (highlighted in red and yellow-green clusters) (5, 6), the living environments, including natural and cultural aspects (shown in green, purple, and orange clusters) (7–9), and policies related to aging population (depicted in blue cluster) (10, 11).

However, fewer than 10% (122 out of 1,309) of studies on population aging have been conducted from a spatial perspective (Figure 1b), which is essential for addressing aging with localized characteristics. Figure 1b categorizes these spatial studies into three main types: spatiotemporal trend of aging development (blue cluster) (12, 13), the allocation of public facilities and services for equitable accessibility (green and purple clusters) for the older adult (14, 15), and the spatial heterogeneity and influencing factors of population aging (red cluster) (3, 16).

Spatial analysis has emerged as a critical methodology for examining geospatial patterns of population aging data and uncovering hidden demographic dynamics. As conceptualized by Haining (17) (p. 4–5), this approach integrates three fundamental components: cartographic modeling, mathematical modeling, and spatial data analytics. The proliferation of geographical information platforms—e.g., ArcGIS, GeoDA (18), and specialized packages in R (19) and Python (20)—has significantly enhanced the application of spatial analysis techniques in demographical studies. This methodological advancement has facilitated innovative investigations into aging patterns, as exemplified by recent research: Wang et al. (21) employed spatial autocorrelation and Bayesian spatio-temporal effect model to explore the dynamic relationship between mainland China’s aging population distribution and medical resources allocation. Similarly, Xu et al. (22) utilized global and local spatial autocorrelation indices and spatial econometric models to examine provincial-level disparities in self-assessed health among older adult population in China.

The spatial dimensions of older adult care provision have become prominent themes in demographical research. Scholars have adopted diverse spatial analytical framework to address service accessibility challenges. Li et al. (23) conducted a spatio-temporal assessment of rural institutional older adult care resource distribution. Li et al. (24) employed provincial panel data to analyze urban–rural equity in older adult care services accessibility. Zhang et al. (25) highlighted the uneven allocation of older people care resource across China’s regions.

Scholarly investigations into spatial patterns of population aging and influencing factors operate across multiple geographical hierarchies, encompassing national (12, 26), regional (16, 27), provincial (28, 29), and municipal (30, 31) levels. These studies use diverse spatial datasets ranging from provincial administrative units (16), to townships records (30) and pixel-level grids (13). The phenomenon of population aging, characterized by demographic theory as “an inevitable part of the transition to lower rates of population growth that follow the demographic transition from high fertility and high mortality to low fertility and low mortality” (32) (p.7), exhibits multifaceted drivers. Empirical studies have identified several determinant categories of population aging. (i) Demographic drivers: Huang et al. (33) established through prefecture level modeling that natural aging, fertility, mortality, and migration collectively reconfigure age structures. (ii) Economic forces: Man et al. (12) revealed significant provincial-level correlations between aging and economic indicators, while Wu and Song also discussed the impacts of economic factors on aging. (iii) Healthcare systems: Iuga et al. (34) demonstrated through cross European Union that health care expenditure and hospital bed availability mediate aging progression rates; Ma et al. (35) investigated the coupling and coordination between the demand of healthcare resources and the older adult population, while Wu et al. (36) revealed the increasing imbalance between supply and demand of older adult care resources in the Yangtze river delta regions of China. (iv) Natural environmental conditions: Lian et al. (37) explored natural environmental factors on aging in China’s mountainous areas; Xu et al. (9) and Chen et al. (6) discussed the close relationship between population aging and air pollution driven mortality. And (v) other factors: except for other economic factors, Zhou et al. (38) also discussed the settlement costs on aging in the Yangtze river delta urban agglomeration. In our research, we examined how demographic, economic, health care, and natural environmental factors impact aging rates and how these influences vary across different scales.

The proliferation of geospatial big data has catalyzed a paradigm shift from singular-data analysis to multisource data fusion, particularly in overcoming the spatial–temporal limitations of conventional statistical sources. While cell phone signaling and point-of interest (POI) datasets have proven effective in population downscaling (39–41), exploring spatial patterns of demographic subgroups (42, 43), and economic data spatialization (44, 45), night time light (NTL) remote sensing has been verified as an valuable data source across multidisciplinary investigations. Andries et al. (46) systematically identified 58 potential research themes of NTL spanning 10 sustainable development domains (p. 12–15), confirming the capacity of NTL data to bridge macroscale statistical indicators with microscale spatial processes. Given our research focus on population aging and its influencing factors encompassing four categories, we specifically contextualize NTL’s applicability within three pertinent domains. The first type pertains to estimation or evaluation of economic indicators such as gross domestic product (GDP) growth rate (47), income (48), unemployment rates (49). The second type relates to population spatialization, for example, simulating population in 1-kilometer grid, informing statistical models to obtain long-term population spatialization results (50). And the third type is about exploring urban functional zones (51), identifying lighting characteristics of public space in urban functional areas (52). These contributions display NTL’s dual role as both a standalone spatial proxy and a synergistic data layer that enhances conventional socioeconomic measurements through geospatial contextualization.

Although aging issues have been extensively studied across China, the spatial pattern and influencing mechanism in multiscale of population in Shaanxi and Gansu provinces remain underexplored. Located in the underdeveloped northwestern China, Shaanxi and Gansu provinces have similar population structures (2) and represent the most aged populations among China’s five northwestern administrative units (alongside Qinghai, Tibet, and Xinjiang). Their selection as study areas is justified by three distinctive attributes. From the geographical aspect, both provinces demonstrate pronounced intraregional diversity: Shaanxi’s “Loess Plateau (north)–Guanzhong Plain (central)–Qinba Mountain Area” (south) structure (53) contrasts with Gansu’s “Hexi Corridor (northwest)–Loess Plateau (central and east)–Qinba Mountain Area (south)–Gannan Plateau (southwest)” configuration (54). Such spatial stratification likely generates subprovincial disparities in aging severity and policy responsiveness. From the political perspective, their strategic roles diverge significantly, Shaanxi functions as the operational core of the Western Development Strategy (55) and anchors the Guanzhong Plain urban agglomeration, while Gansu serves as the critical terrestrial corridor of the Belt and Road Initiative (56). These contrasting national positions may differentially influence resource allocation for older adult care systems. In socioecological aspect, two compounding factors emerge: first, the two provinces both have concentrated ethnic minority settlements, and policies need to consider ethnic factors, which increase the complexity of aging governance; and second, the soil erosion area of the Loess Plateau in northern Shaanxi and arid area of the Hexi Corridor in Gansu have fragile ecological capacity (57), which drive youth outmigration so that the proportion of left behind older adult is increasing.

The remainder of this research is organized as follows. Section 2 outlines materials and methods, including data collection and preprocessing, spatial autocorrelation (SA) analysis, and multiscale geographical weighted regression (MGWR). Section 3 presents the globe and local spatial patterns of aging rates in the Shaanxi-Gansu region for the years 2010 and 2020. Section 4 explores and visualizes the multiscale impacts of demographic, economic, healthcare, and natural environmental factors on aging. Finally, section 5 concludes with a summary of the findings and discusses their policy implications.

## 2 Materials and methods

### 2.1 Study area

**Figure 2:**
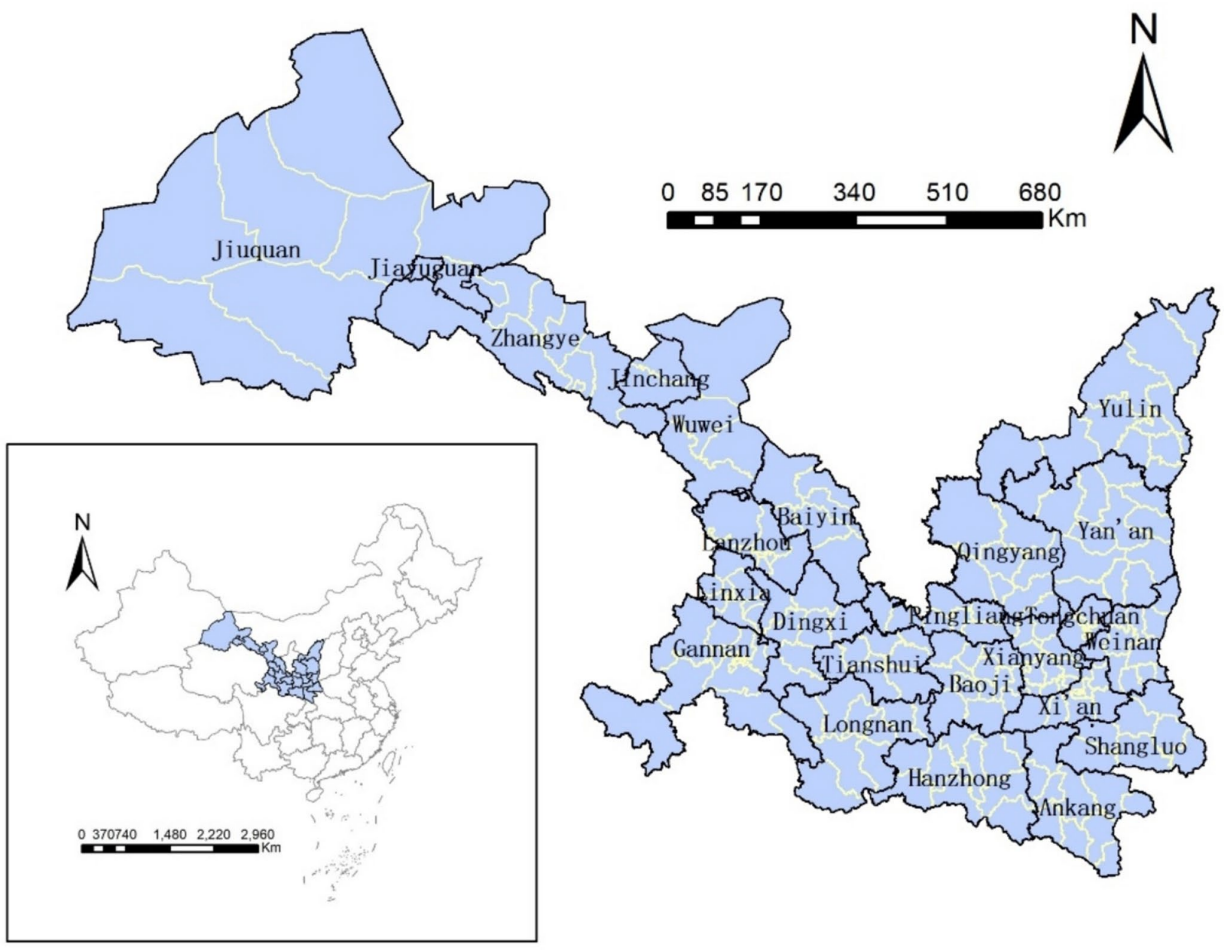
The Shaanxi-Gansu region.

Shaanxi and Gansu provinces are located in the northwestern China, with Shaanxi in the middle reaches of the Yellow River and Gansu to its west, they also feature complex geographical environments, including the Loess Plateau and Qinling Mountains. Since there were no significant changes in county-level administrative divisions between 2010 and 2020, this study uses counties as the basic unit of analysis, totaling 194 units. As illustrated in Figure 2, the prefecture-level administrative divisions are outlined with solid black lines, while the county-level administrative divisions within each prefecture are marked with solid yellow lines.

### 2.2 Data and variables

This study utilized population and influencing factors datasets derived from the sixth (2010) and seventh (2020) national population censuses, as well as the “Shaanxi Statistical Yearbook” and “Gansu Statistical Yearbook” (2010 and 2020).

The focus of this research was the aging rate, defined as the percentage of the population aged 65 years and older, which served as the primary variable of interest. According to the United Nations (58) (p. 7), a country or region is considered “aged” when the proportion of individuals aged 65 and above exceeds 7% of the total population. Organisation for Economic Co-operation and Development (OECD)/World Health Organization (WHO) (59) further classified aging levels based on the aging rate: 7–14% is considered “mild aging,” 14–21% “moderate aging,” and over 21% “severe aging.”

Population aging is influenced by various, including demographic (60), economic (61), healthcare (62), and natural environmental factors (30). This study analyzed the impact of these four categories of factors on aging rates. We originally collected 11 variables across these categories: demographic factors (birth rate, mortality rate, and proportion of permanent population); economic factors (GDP, per capita GDP, per capita disposable income of urban residents, and net income of rural residents); healthcare factors (number of health institutions and number of community service institutions and facilities); natural environmental factors: (days of good air quality and per capita park green area).

### 2.3 Research methods

#### 2.3.1 Data processing

This study employed county-level administrative units as the fundamental research scale. Although comprehensive datasets were collected for demographic variables (i.e., birth rate, mortality rate, proportion of permanent population) and economic indicators (i.e., GDP, per capita GDP), partial missing values were identified in the records of health institutions and community healthcare facilities. To address these data gaps, we leveraged night time light (NTL) data as a supplementary proxy based on their proven ability to reflect the intensity of human activities (63) including economic activities. Empirical evidence demonstrates that NTL intensity has medium to strong positive correlation with enterprise density and income levels (64), and serves as a reliable substitute for conventional economic indicators in countries and regions with very poor quality or missing data (65). Given the established positive relationship between economic development level and healthcare services supply (66) and healthcare expenditure (67), we hypothesize a correlation chain: NTL intensity → economic development level → healthcare service provision. This principle justifies utilizing NTL data to impute missing values for the two variables: the number of health institutions (NoHI) and the number of community service institutions and facilities (NoCSIF). The imputation procedure incorporated NTL datasets from the DMSP-OLS and NPP-VIIRS (2010–2020) corrected by Zhong et al. (68) through tree operational steps.

Step 1, Spatial aggregation: NTL values in grid cells were aggregated to counties and prefecture-level cities by summing all NTL values within each county and prefecture-level city.

Step 2, Weight coefficient derivation: County-specific NTL weights were computed as the ratio of individual county NTL intensity to total prefecture-level city NTL intensity.

Step 3, Missing value estimation: For counties with missing NoHI/NoCSIF records, values were imputed by multiplying reported prefecture-level city totals by corresponding county NTL weights.

To ensure the independence of variables, we conducted a multicollinearity analysis (results are summarized in Supplementary Table S1) and retained only those with variance inflation factors (VIF) less than 9. Table 1 lists the eight variables included in the final model.

**Table 1:** Influencing factors of population aging employed in model.

Types	Name	Representation in model
Demographic factors	Birth Rate	BIR
	Mortality Rate	MOR
	Proportion of Permanent Population	PoPP
Economic factors	per capita GDP	pcGDP
Healthcare factors	Number of Health Institutions	NoHI
	Number of Community Service Institutions and Facilities	NoCSIF
Environmental factors	Days of Good Air Quality	DoGAQ
	per capita Park Green Area	pcPGA

#### 2.3.2 Spatial autocorrelation analysis

Spatial autocorrelation analysis consists of global and local autocorrelation methods. In this study, we employed the global Moran’s *I* index (69) and Getis-Ord $G_{i}^{*}$ index (70) to examine the spatial clustering patterns of population aging in the Shaanxi-Gansu region.

The Moran’s *I* index typically ranges from −1 to 1. A value between 0 and 1 indicates positive spatial autocorrelation, meaning similar values are more likely to cluster together. A value between −1 and 0 indicates negative spatial autocorrelation, implying dissimilar values are more likely to be spatially adjacent. A value close to 0 suggests a random spatial distribution with no discernible spatial pattern.

The local indicator of spatial association, specifically the standardized Getis-Ord $G_{i}^{*}$ index (70) used in this paper, identifies local spatial clusters of high or low values. A significant positive $G_{i}^{*}$ indicates that both the focal region and its surrounding areas have high attribute values, forming a “hot spot” or high–high cluster. Conversely, a significant negative $G_{i}^{*}$ value suggests that both the focal region and its surrounding areas have low attribute values, forming a “cold spot” or a low–low cluster.

#### 2.3.3 Multiscale geographically weighted regression (MGWR)

As an upgraded version of the geographical weighted regression (GWR) model (71) which furnishes different estimations for the coefficients of the same independent variable across the research areas, the MGWR (72) can identify the specific spatial range within which each explanatory variable exerts its influence, and the ranges varies with variables, which implies that the independent variables affect the dependent variable in multi-scales.

The MGWR model is represented as:

$$y_{i}=\beta_{bw_{0}}\left(u_{i},v_{i}\right)+\underset{k}{\sum }\beta_{bw_{k}}\left(u_{i},v_{i}\right)x_{ik}+\varepsilon_{i}\text{,}$$

where $\beta_{bw_{k}}$ represents the bandwidth of the *k*th variable, $y_{i}$ is the *i*th observation of the dependent variable, $x_{ik}$ is the *i*th observation of the *k*th explanatory variable, $\left(u_{i},v_{i}\right)$ is the spatial coordinate of location *i*, $\beta_{bw_{0}}\left(u_{i},v_{i}\right)$ is the intercept term and $\beta_{bw_{k}}\left(u_{i},v_{i}\right)$ is the regression coefficient of unit *i*, and $\varepsilon_{i}$ is an error term that follows a normal distribution. We employed the MGWR model not only to explore the different intensities of a variable impacting on different areas, but also to explore the impacting ranges of different variables on aging rates.

## 3 Results and analysis

### 3.1 The spatiotemporal pattern of population aging

From 2010 to 2020, Shaanxi and Gansu provinces experienced notable increases in population aging, as shown in Table 2. In Shaanxi, the average population aging rate rose from 8.53 to 13.32%, while in Gansu, it increased from 8.23 to 12.58%. Additionally, the range of aging rates expanded in both provinces: from 6.74 to 15.88% in Shaanxi and from 6.99 to 12.55% in Gansu. These trends highlight a significant acceleration in population aging over the decade, accompanied by growing inter-regional disparities in the aging process.

**Table 2:** Some summary statistics of aging rates of Shaanxi and Gansu provinces from 2010 to 2020.

Province	Statistics	Aging rates of 2010	Aging rates of 2020
Shaanxi province	Max	12.01	23.42
	Min	5.27	7.54
	Range^*^	6.74	15.88
	Mean	8.53	13.32
Gansu province	Max	10.63	18.03
	Min	3.64	5.48
	Range	6.99	12.55
	Mean	8.23	12.58

* Range = Max − Min.

#### 3.1.1 The global spatial pattern

The Moran’s *I* values, measuring global clustering of the aging rates in Shaanxi and Gansu in 2010 and 2020, are shown in Table 3, and all values indicate moderate spatial autocorrelation. More specifically, the Moran’s *I* values for the aging rates in Shaanxi province in 2010 and 2020 are 0.405 and 0.430, respectively, indicating significant global spatial clustering characteristics. The Moran’s *I* values for the aging rates in Gansu province are 0.318 and 0.402, respectively, which are slightly lower than those of Shaanxi province, but also show significant global spatial clusters.

**Table 3:** Global Moran’s *I* estimation of aging rates in the Shaanxi-Gansu region in 2010 and 2020.

Region	Year	Moran’s *I*	*Z*-score	*p*-value
Shaanxi province	2010	0.405	6.969	0.000***
	2020	0.430	7.415	0.000***
Gansu province	2010	0.318	4.605	0.000***
	2020	0.402	5.772	0.000***
Shaanxi-Gansu region	2010	0.385	8.920	0.000***
	2020	0.442	10.220	0.000***

Significance level: *0.1, **0.05, ***0.01.

**Figure 3:**
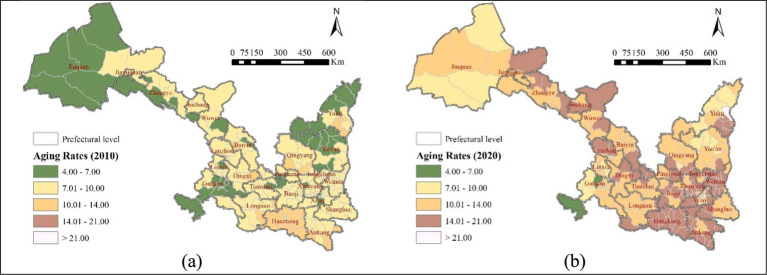
Distribution of aging rates in the Shaanxi-Gansu region: **(a)** spatial distribution of aging rates in 2010; and **(b)** spatial distribution of aging rates in 2020.

Figure 3 illustrates the spatial distribution of aging rates in the Shaanxi-Gansu region, revealing significant moderate spatial autocorrelation. The spatial pattern of aging rates aligns closely with the geographical features of the two provinces: those relatively low aging areas are predominantly distributed in northwestern Gansu, northern Shaanxi, and the ethnic gathering places in southwestern Gansu; while high aging clusters are in southern Shaanxi and Gansu within the Qinba mountain where experiences substantial youth outmigration.

Figure 3 also shows a rapid increase in aging rates across the Shaanxi-Gansu region from 2010 to 2020, accompanied by widening regional disparities. Notably, some counties in central (e.g., Fufeng and Qishan counties in Baoji) and northern (e.g., Jia county in Yulin) Shaanxi, and central (e.g., Minqin county in Wuwei) Gansu experienced significantly higher growth rates of aging than other areas. In contrast, Ethnic minority areas (e.g., Hezuo and Maqu counties in the Gannan Tibetan Autonomous Prefecture, Guanghe county in the Linxia Hui Autonomous Prefecture) in southwest Gansu, and central areas in Shaanxi (e.g., Lianhu and Baqiao districts in Xi’an) exhibited significantly lower aging rates.

Except the ethnic gathering places in southwestern Gansu, almost all areas’ aging rates had passed the internation threshold 7% by 2020, and some areas had even entered a moderately aged society. These include southern areas (e.g., Hanzhong, Ankang) and part central (Weinan) and northern areas (southeastern Yulin) of Shaanxi, Similarly, some central and eastern regions (Dingxi, Pingliang, Tianshui, Lanzhou) in Gansu have also transitioned into a moderately aged society.

#### 3.1.2 The local spatial pattern

**Figure 4:**
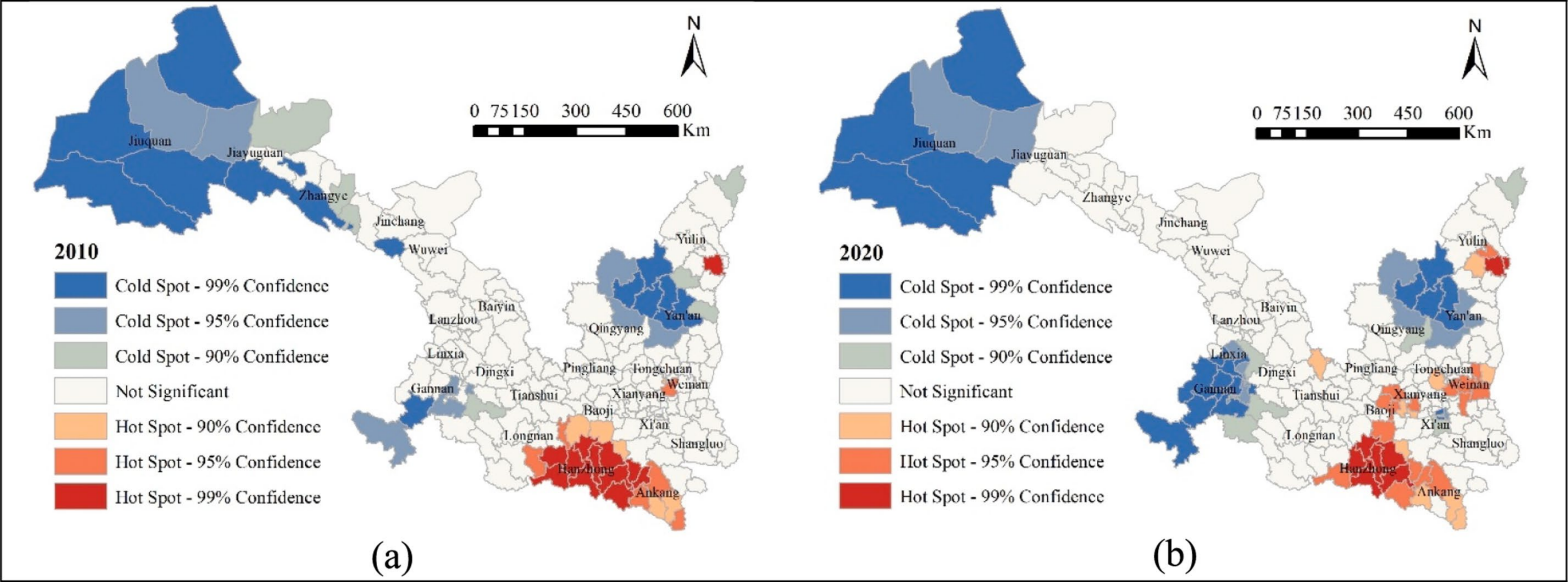
Evolution of the local spatial pattern of population aging in the Shaanxi-Gansu region: **(a)** local clusters of aging rates in 2010; and **(b)** local clusters of aging rates in 2020.

Figure 4 presents the spatiotemporal evolution of aging rate clusters in the Shaanxi-Gansu region during 2010–2020 using the $G_{i}^{*}$ statistics. The $G_{i}^{*}$ values are categorized into seven significant levels: cold spots with three levels of significance (99, 95, and 90%), hot spots with three levels of significance (99, 95, and 90%), and insignificant areas. For clarity, these categories are simplified as: cold spots (99%), moderate-cold spots (95%), sub-cold spots (90%), insignificant zones, sub-hot spots (90%), moderate-hot spots (95%), and hot spots (99%). Spatial patterns reveal distinct clustering dynamics: low-aging clusters (blue) predominantly occupy northwestern Gansu and Shaanxi, and southwestern Gansu; while high-aging clusters (red) concentrate in southern areas, particularly the Qinba mountains, exhibiting persistence of youth outmigration across the decade.

Between 2010 and 2020, notable changes occurred in the distribution of hot spots and cold spots. In areas with low-aging rate clusters, some cold spots and sub-cold spots in northwestern Gansu (Jiuquan and Zhangye) disappeared. In contrast, cold and sub-cold spots in partly southwestern (Gannan Zang Autonomous Prefecture) Gansu expanded and spread to neighboring areas. Meanwhile, cold spots in northwestern Shaanxi (Yanan) and eastern Gansu (Qingyang) remained largely unchanged, a small sub-cold spot emerged in Xi’an. The shrink of low-aging clusters in northwestern Gansu presents the decreased fertility intension with urbanization in these areas; the nearly unchanged low-aging clusters are located in the Loess Plateau between boundaries of the two provinces where people still have traditional fertility concept; the expanded low-aging areas in ethnic minority areas of southwestern Gansu and districts in Xi’an (Lianhu, Xincheng, Yanta) may be the results of the relaxation of fertility policy issued in 2015 which have limited impacts on Han population (73).

In regions with high-aging rate clusters, southern areas of both provinces have little changes, middle-hot spots and sub-hot spots appeared in some central areas (Weinan and Baoji) of Shaanxi, and the southeastern Yulin which locates in northern Shaanxi experienced an expansion of hot spots. High-aging areas in southern Shaanxi-Gansu are located in Qinba mountain areas where young population consistently out-migrated; those newly emerged high-aging areas are largely located in regions experienced rapid urbanization progress (74).

In summary, most areas in southern Shaanxi-Gansu located within Qinba moutains continue experiencing rapid population aging because of sustained youth outmigration, while emerging high-aging areas in parts of central and northern Shaanxi and the diminished low-aging areas in northwestern Gansu experienced accelerated urbanization. In contrast, low-aging areas are mainly concentrated in ethnic minority gathering areas in southwestern Gansu and some districts in Xi’an city.

### 3.2 Modeling the relationship between population aging and its influential factors

In this section, we employed five modeling techniques—Ordinary Least Squares (OLS), spatial lag model (SLM) (75), spatial error model (SEM) (75), Geographically Weighted Regression (GWR), and Multiscale Geographically Weighted Regression (MGWR)—to examine the factors influencing population aging. OLS is the baseline model, SLM and SEM are global spatial regression models, while GWR and MGWR are local spatial regression models. We compared these models based on goodness of fit, residual autocorrelation and normality.

#### 3.2.1 The comparison of goodness-of-fit

As shown in Table 4, the MGWR model exhibited the lowest residual sum of squares (RSS) and Akaike information criterion corrected (AICc) values, along with the highest *R*^2^ and adjusted *R*^2^ among the five models. These results indicate that the MGWR model provides the best fit.

**Table 4:** Fitting comparisons of OLS, SLM, SEM, GWR, and MGWR models.

	2010					2020				
Models	OLS	GWR	SLM	SEM	MGWR	OLS	GWR	SLM	SEM	MGWR
RSS	106.24	52.86	193.99	165.75	**47.76**	102.47	49.97	622.04	499.58	**37.55**
AICc	454.93	401.53	581.50	561.18	**375.19**	447.93	362.63	811.29	785.38	**332.07**
*R* ^2^	0.45	0.73	0.57	0.63	**0.75**	0.47	0.74	0.64	0.71	**0.81**
Adj. *R*^2^	0.43	0.66	0.55	0.61	**0.70**	0.45	0.69	0.62	0.70	**0.76**

The bold values mean the best model fitting.

#### 3.2.2 The comparison of residuals

The spatial autocorrelation of model residuals is a crucial indicator for model validity. As presented in Table 5, the MGWR model produced non-significant or lowest values for residuals, while OLS, SLM, and GWR present the significant SA values for residuals. The residuals of SEM are non-significant because the model includes spatial autocorrelation in the error term in the modeling process. These results suggest that the residuals generated by the MGWR most conform to the independent assumption of the error term of the regression model.

**Table 5:** Residual spatial autocorrelation of five regressive models.

Year	Models	Moran’s *I*	*Z*-score	*p*-value
2010	OLS	0.315	7.308	0.000***
	GWR	0.089	2.161	0.031**
	SLM	0.089	2.008	0.022**
	SEM	−0.021	−0.336	0.632
	MGWR	0.062	1.543	0.123
2020	OLS	0.349	8.092	0.000***
	GWR	0.140	3.310	0.001***
	SLM	0.105	2.337	0.009***
	SEM	0.016	0.443	0.329
	MGWR	0.079	1.919	0.055*

Significance level: *0.1, **0.05, ***0.01.

Table 6 presents the results of residual normality test. The analysis reveals that SLM, SEM, and MGWR produced normal or approximate normal distribution. In contrast, the *p*-values for the OLS and GWR models suggest rejection of the null hypothesis of residual normality.

**Table 6:** The results of residual normality test.

Year	Models	*W*	*p*-value
2010	OLS	0.945	0.000***
	GWR	0.971	0.036**
	SLM	0.990	0.224
	SEM	0.987	0.082*
	MGWR	0.991	0.277
2020	OLS	0.961	0.000***
	GWR	0.985	0.044**
	SLM	0.994	0.632
	SEM	0.992	0.373
	MGWR	0.993	0.342

Significance level: *0.1, **0.05, ***0.01.

In summary, MGWR model presents the best performances. The global model constructed using the OLS, SLM, and SEM failed to adequately address the spatial heterogeneity of aging patterns, which is coincident with the results in subsection 3.1.2 that the aging in Shaanxi-Gansu region have spatial heterogeneity. Although the GWR model accounts for spatial heterogeneity in population aging, its use of a uniform bandwidth for analyzing influencing factors overlooks the inherent differences among these factors, and thus also failed to describe the data generating process. In contrast, the MGWR model offers significant advantages over the GWR model: it not only enables spatial heterogeneity in parameter estimation but also generates distinct optimal bandwidth values for the relationships between the response variable and each predictor variable. This capability enables the simulation of spatial processes operating at different scales (72).

## 4 Discussion

**Figure 5:**
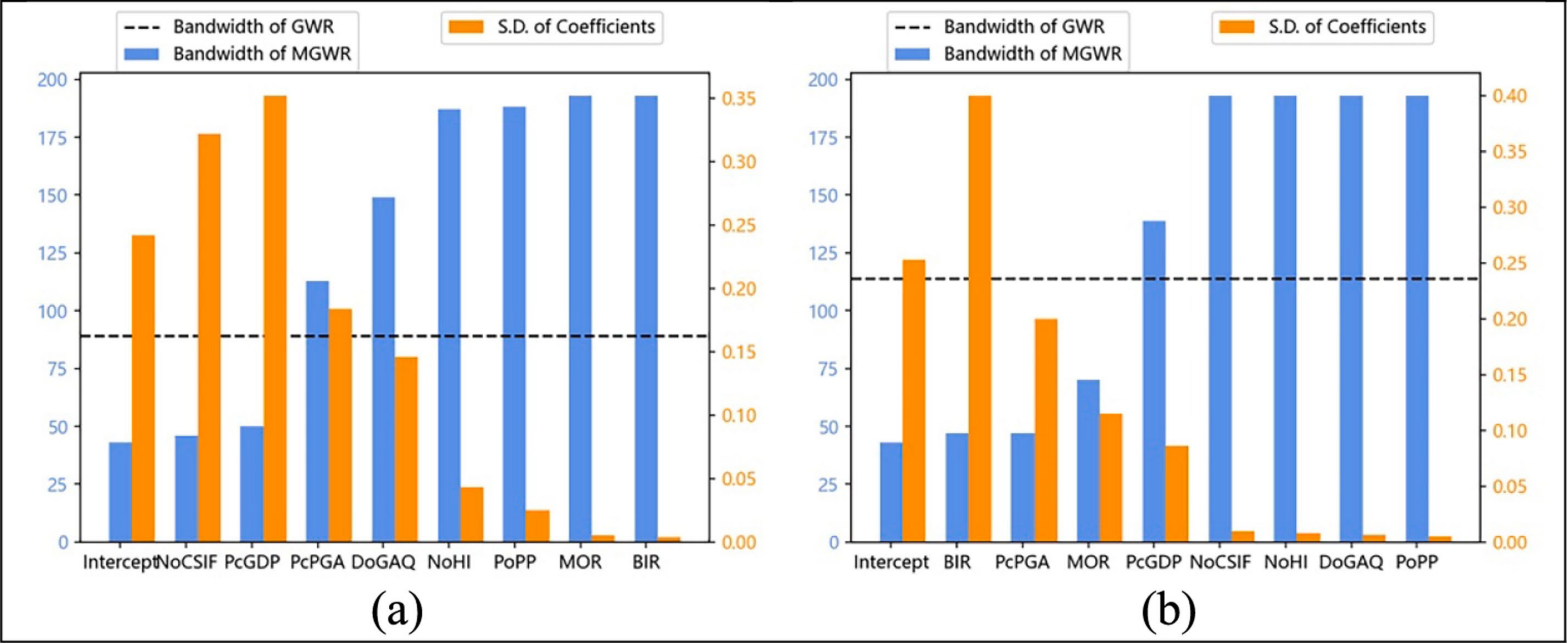
The optimal bandwidth generated by MGWR and GWR and the standard deviation of MGWR parameter estimates (The horizontal axis represents the selected variables, the vertical axis on the left represents the bandwidth of MGWR, and the vertical axis on the right represents the standard deviation of parameter estimation generated by the MGWR): **(a)** the bar chart of 2010, and **(b)** the bar chart of 2020.

This section discusses both the intensity and spatial extent of factors affecting aging rates. Table 7 presents descriptive statistics of the estimated model coefficients, while Figure 5 visualizes the optimal bandwidths derived from the MGWR and GWR models. In Figure 5, orange bars represent the standard deviations of MGWR parameter estimates, and the black dotted line indicates the GWR optimal bandwidths. Variables with larger bandwidths exhibit broad-scale spatial effects and lower spatial heterogeneity, resulting in smaller standard deviations in parameter estimates. In contrast, variables with smaller bandwidths reflect localized effects, leading to greater variability in local parameter estimates.

**Table 7:** Descriptive statistical results of the MGWR model.

Year	Variable	Mean	STD	Min	Median	Max
2010	BIR	−0.470	0.004	−0.477	−0.470	−0.457
	MOR	0.160	0.005	0.141	0.162	0.170
	PoPP	−0.469	0.025	−0.491	−0.484	−0.412
	PcGDP	−0.134	0.352	−0.798	−0.167	0.355
	NoHI	0.095	0.043	0.064	0.074	0.250
	NoCSIF	0.100	0.322	−0.505	0.004	0.656
	DoGAQ	0.243	0.146	0.024	0.349	0.371
	PcPGA	−0.100	0.184	−0.380	−0.104	0.165
2020	BIR	−0.273	0.400	−0.831	−0.260	0.409
	MOR	0.136	0.115	−0.269	0.151	0.371
	PoPP	−0.508	0.005	−0.519	−0.510	−0.495
	PcGDP	0.032	0.086	−0.157	0.032	0.145
	NoHI	−0.052	0.008	−0.071	−0.048	−0.045
	NoCSIF	−0.077	0.010	−0.106	−0.072	−0.068
	DoGAQ	0.131	0.007	0.125	0.128	0.153
	PcPGA	−0.105	0.200	−0.455	−0.130	0.404

Figure 5 shows that in 2010, demographic factors such as birth rate, mortality rate, and proportion of permanent population exhibited global-scale effects on population aging, indicating consistent impacts across counties and districts. In contrast, per capita GDP demonstrated local-scale influences, suggesting significant spatial heterogeneity in economic effects. Medical factors displayed mixed patterns: the number of health institutions (NoHI) had global effects, while the number of community service institutions and facilities (NoCSIF) showed local variations, highlighting the uneven impacts of community service development across counties and districts. Environmental factors operated at a regional scale, demonstrating moderate spatial heterogeneity in their influence on aging patterns.

**Figure 6:**
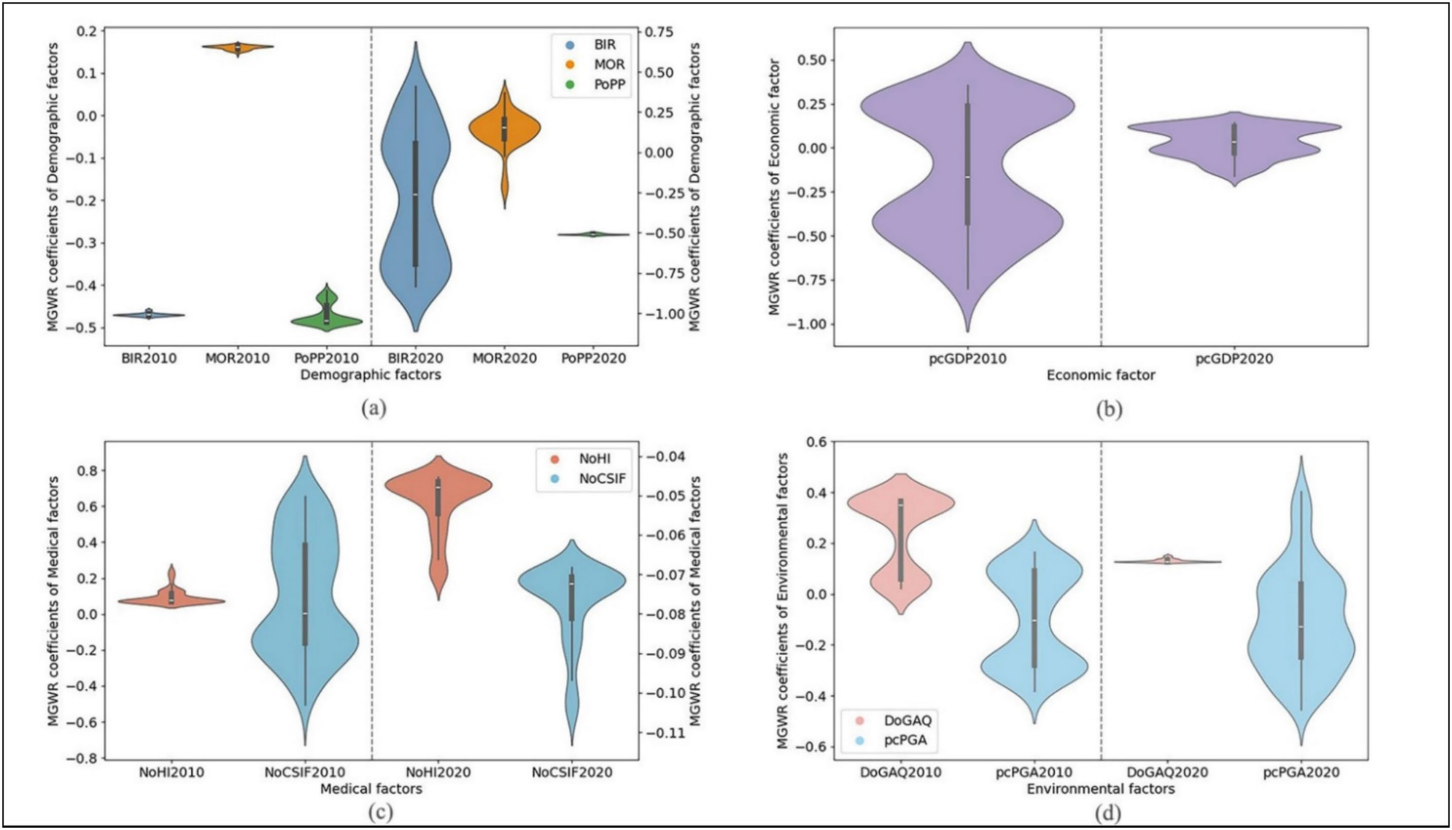
Violin plot of MGWR coefficients of influencing factors. For subfigures **(a–d)**, the coefficients of 2010 correspond to the left vertical axis, and the coefficients of 2020 correspond to the right vertical axis.

By 2020, notable shifts in spatial patterns emerged. While the proportion of permanent population continued to exert a global effect on aging, birth and mortality rates shifted to local scale influences, reflecting increased population mobility and growing demographic diversity across areas. The influence of per capita GDP expanded to a regional scale, suggesting economic spillover effects among adjacent districts and counties. Medical factors maintained their global impact, likely due to the standardization of healthcare and welfare facilities across areas. Among environmental factors, per capita park green area (pcPGA) exhibited reduced spatial influence, whereas days of good air quality (DoGAQ) demonstrated broader regional consistency. This indicates that air quality effects became more uniform across regions, contrasting with the localized and spatially heterogeneous impacts of urban greening on aging patterns. The influencing intensities of each factor are shown in Figure 6. The successive subsections demonstrate how factors influence aging and their policy implications.

### 4.1 Multiscale spatial heterogeneity of demographic factors’ impacts on population aging

Figure 6a presents a violin plot of MGWR coefficients for demographic factors, with the 2010 and 2020 coefficients corresponding to the left and right vertical axes, respectively. Among these factors, birth rate (BIR) exhibited the largest variation in coefficient, followed by mortality rate (MOR). In 2010, the coefficients for BIR and proportion of permanent population (PoPP) were similar, both between −0.5 and −0.4 and showing distinctly negative effects, while MOR which ranged from 0.1 to 0.2 exhibited positive effects. By 2020, BIR coefficients showed conspicuous variability with some shifting to positive values, showing that BIR were positively correlated with aging rates in some areas. MOR coefficients, although partially negative, predominantly clustered around 0.15, while PoPP coefficients remained stable around −0.5, showing minimal change from 2010.

#### 4.1.1 The impact of birth rate (BIR)

**Figure 7:**
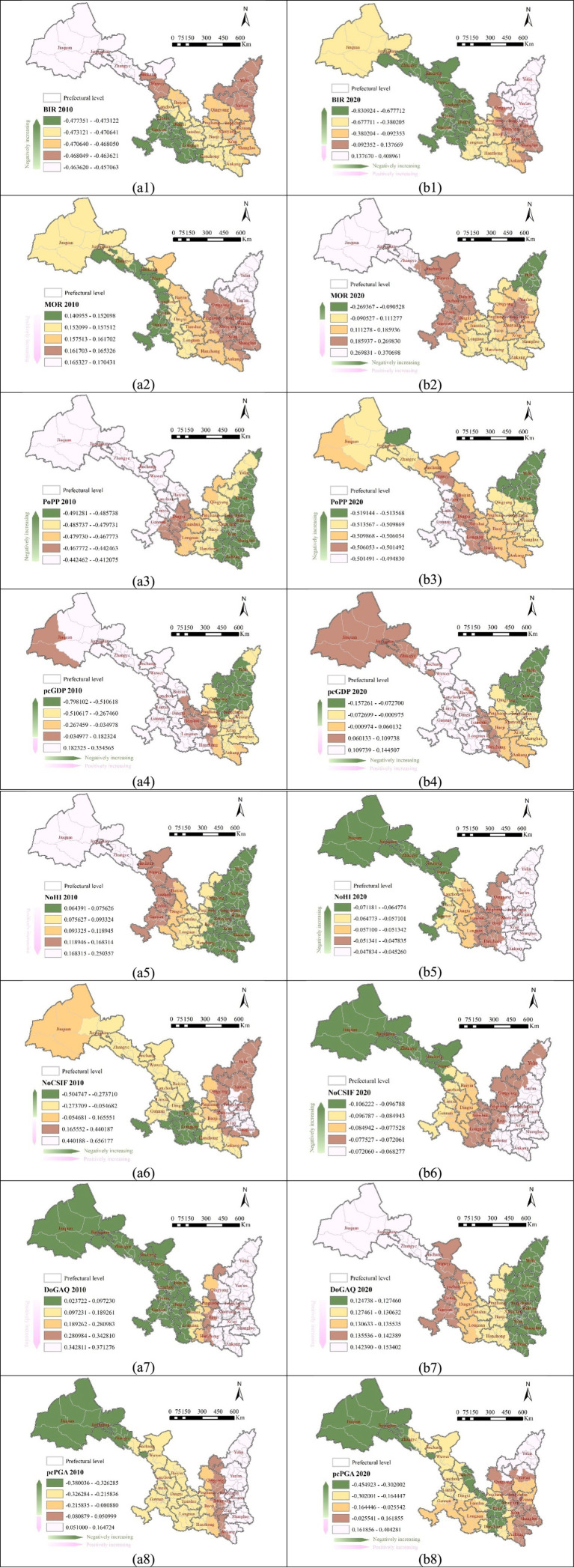
Impacting ranges and intensities of four types of factors: **(a1-b3)** demographic factors; **(a4-b4)** the economic factor; **(a5-b6**) medical factors; **(a7-b8)** environmental factors.

The BIR predominantly shows a negative correlation with population aging. In 2010, BIR exhibited global-scale effects on aging (Figure 5), with particularly strong negative impacts observed in southwestern (Gannan Tibetan and Linxia Hui Autonomous Prefecture), and central-southern (Dingxi and Longnan) Gansu (Figure 7a1). By 2020, BIR’s influence shifted to a local scale (Figure 5), becoming more pronounced in central and southern Gansu province. However, in northern Shaanxi province, BIR’s effect reversed from negative to positive, though with reduced intensity (Figure 7b1).

#### 4.1.2 Mortality rate (MOR)

The MOR shifted from a global-scale impact in 2010 to a local-scale influence by 2020 (Figure 5). In 2010, MOR exhibited strong effect in northern Shaanxi (Yulin and Yan’an), while its influence was relatively weak in northern-central Gansu (particularly in Zhangye, Wuwei, Lanzhou), and the two ethnic minority autonomous prefectures in southwestern Gansu (Figure 7a2). By 2020, MOR’s impact became negative in northern and southern Shaanxi, while its influence positively intensified in the northwestern Gansu (Jiuquan, Jiayuguan, Zhangye, Figure 7b2). This shift highlights an increasing regionalization of MOR’s impact on aging.

#### 4.1.3 Proportion of permanent population (PoPP)

The PoPP consistently exerted a negative, global-scale influence on population aging in both 2010 and 2020 (Figure 5). In 2010, PoPP had strong effects across Shaanxi province, while its influence was relatively weaker in most areas of Gansu province, except eastern (Qingyang, Pingliang) and southern (Tianshui, Longnan) areas in Gansu which are neighboring to Shaanxi (Figure 7a3). By 2020, the overall intensity of Popp’s impact increased, with particularly notable growth in northwestern (Jiuquan, Jiayuguan, Zhangye) and the two ethnic minority autonomous prefectures Gansu.

The relationships between demographical factors and aging rates reveal two key implications. First, the aging rate in Gansu demonstrates greater sensitivity to birth rate fluctuations compared to Shaanxi. Specifically, a 1% increase in birth rate would yield a more substantial reduction in aging rate in Gansu than in its neighboring province. This differential responsiveness suggests that policy interventions aimed at incentivizing childbirth may generate more significant demographic dividends in Gansu. Second, the proportion of permanent population has exerted consistently global negative effects on aging rates across Shaanxi-Gansu region through the study period. This persistent pattern underscores the necessity for sustained policy efforts to enhance regional attractiveness to younger migrants through improved living conditions and employment opportunities.

### 4.2 Multiscale spatial heterogeneity of economic factor’s impacts on population aging

Figure 6b displays a violin plot MGWR coefficients of the economic factor. In 2010, per capita GDP (pcGDP) coefficients exhibited significant variation, encompassing both positive and negative values. The distribution showed two distinct clusters: positive coefficients centered around 0.25 and negative coefficients around −0.45, highlighting pronounced economic disparities among cities and counties and their contrasting effects on aging. By 2020, the coefficient distribution narrowed considerably, primarily falling between 0.00 and 0.25, suggesting a substantial reduction in inter-regional economic disparities over the decade.

The spatial influence of pcGDP shifted from a local scale in 2010 to a regional scale by 2020 (Figure 5). In 2010, Northern Shaanxi (Yan’an and Yulin) and eastern Gansu (Qingyang) displayed notably stronger negative pcGDP effects on aging compared to other areas (Figure 7a4). By 2020, the overall regional influencing intensity (in absolute value) of pcGDP on aging rate weakened (Figure 7b4).

With the economic development, aging rates became progressively insensitive to pcGDP across areas, and the range of economic effects on aging extended geographically. According to the influencing trend of economic development on aging rate in the two provinces, with each 1% increment of pcGDP, expenditures on aging care and services may be more allocated to central-southern Gansu, followed by northern areas of Gansu. Concurrently, eastern Gansu and Shaanxi (particularly those northern areas) require proactive fiscal preparedness to address imminent aging-induced demographic pressures.

### 4.3 Multiscale spatial heterogeneity of medical factors’ impacts on population aging

Figure 6c displays a violin plot of MGWR coefficients for medical factors, with 2010 coefficients referenced to the left vertical axis and 2020 coefficients to the right. Between 2010 and 2020, significant changes occurred in the coefficients for both NoHI and NoCSIF, particularly for NoHI. In 2010, the two factors exhibited distinct coefficient distributions: NoHI coefficients were centered around 0.1, while NoCSIF coefficients were widely dispersed, spanning both positive and negative values. By 2020, NoHI coefficients shifted entirely from positive to negative, indicating a fundamental shift in its relationship with aging. Meanwhile, NoCSIF coefficients became more narrowly distributed and uniformly negative, and significantly weaker in magnitude compared to their 2010 levels.

#### 4.3.1 Number of health institutions (NoHI)

The NoHI maintained a global-scale influence in both 2010 and 2020, but its directional impact completely reversed during this period, transforming from totally positive to totally negative (Figure 5). In 2010, NoHI exhibited the strongest positive effects in northwestern Gansu (Jiuquan, Jiayuguan, and Zhangye), while its influence was notably weak across Shaanxi province (Figure 7a5). By 2020, the intensity of its influence decreased across all regions, with the most pronounced decreases observed in Gansu province, particularly in northwestern Gansu (Jiuquan, Jiayuguan, Zhangye, Jinchang, and Wuwei) (Figure 7b5).

#### 4.3.2 Number of community service institutions and facilities (NoCSIF)

The NoCSIF exhibited a spatial influence shift from a local scale in 2010 to a global scale by 2020 (Figure 5). In 2010, its impact on aging included both positive and negative effects, with the strongest influence concentrated in the intersected areas of central-eastern-southern Shaanxi (Xianyang, Weinan, Tongchuan, and Xi’an). Areas with weak influence were widely distributed across both provinces (Figure 7a6). By 2020, NoCSIF’s influence became uniformly negative, with a substantial decrease in intensity across all regions. A notable shift from positive to negative influence was observed in most areas in Shaanxi province. Similarly, central-southwestern-southern Gansu (Dingxi, Ganan, Tianshui, and Longnan) experienced significant reductions in influence intensity (Figure 7b6).

From 2010 to 2020, both provinces demonstrated substantial growth in healthcare infrastructure, with Gansu experiencing 156 and 498% increases in the number of health institutions (NoHI) and community service institutions and facilities (NoCSIF) respectively, while Shaanxi recorded 152 and 421% growth for these same metrics. The dramatic NoCSIF expansion across Gansu-Shaanxi region facilitated more equitable spatial distribution of community healthcare resources, suggesting a scale transition in their demographic influence—from localized to global-level impacts on aging patterns. In contrast, NoHI maintained consistent global-scale effects throughout the study period. Despite quantitative improvements, the diminished efficacy of these facilities in impacting aging rates underscores the necessity for local governments to optimize allocations over mere numerical expansion.

### 4.4 Multiscale spatial heterogeneity of environmental factors’ impacts on population aging

Figure 6d presents a violin plot of MGWR coefficients for environmental factors, highlighting significant changes of DoGAQ between 2010 and 2020. In 2010, DoGAQ coefficients displayed bimodal distribution with primary concentrations around 0.05 and 0.35, and also with some negative values, indicating both positive and negative influences on aging. The pcPGA coefficients were clustered around 0.1 and −0.3, indicating a bidirectional impact pattern. By 2020, DoGAQ coefficients shifted to exclusively positive values with reduced intensity, while pcPGA coefficients became more dispersed, predominantly within the negative range.

#### 4.4.1 Days of good air quality (DoGAQ)

The DoGAQ consistently exerted a positive influence on aging in both 2010 and 2020, with its spatial influence expanding from a regional to a global scale (Figure 5). In 2010, Shaanxi province experienced predominantly high-intensity impacts, in contrast to the generally low-intensity effects observed across most areas in Gansu province (Figure 7a7). By 2020, although the overall impact intensity decreased significantly, certain regions displayed localized intensity increases. Notably, northwestern Gansu (Jiuquan, Jiayuguan, Zhangye, and Jinchang) showed strengthened aging effects, whereas Shaanxi province experienced a widespread reduction in impact intensity (Figure 7b7).

#### 4.4.2 Per capita park green area (pcPGA)

The pcPGA exhibited influence patterns distinctly different from DoGAQ, showing both positive and negative effects while shifting from a regional to a local spatial influence (Figure 5). In 2010, high-intensity negative impacts were concentrated in northwestern Gansu (Jiuquan, Jiayuguan, and Zhangye), whereas positive effects were observed in northern-central-eastern-southeastern Shaanxi (Yulin, Yan’an, Tongchuan, Weinan, and Shangluo) (Figure 7a8). By 2020, the overall impact intensity increased but became more localized. Specifically, northwestern Gansu (Jiuquan and Jiayuguan), southwestern Shaanxi (Baoji and Hanzhong) experienced intensified negative impacts, while northern Shaanxi (Yulin) continued exhibiting positive effects. Unlike the relatively stratified pattern observed in 2010, the 2020 spatial distribution of pcPGA impacts showed notably increased heterogeneity (Figure 7b8).

Environmental factors analyzed in this study comprise two categories: natural conditions represented by annual days of good air quality (DoGAQ) and human-modified environments quantified through per capita park green area (pcPGA). While DoGAQ exhibited spatially uniform associations with aging rates throughout the study period, pcPGA demonstrated geographically heterogeneous impacts. Notably, pcPGA emerges as a more readily modifiable parameter than DoGAQ for short-term policy interventions. Our regression analysis reveals that a 1% increase in pcPGA corresponds with the most substantial reductions in aging rates across three distinct regions: (i) northwestern Gansu (Jiuquan, Jiayuguan, Zhangye), (ii) eastern Gansu (Baiyin), and (iii) southwestern Shaanxi, followed by moderate effects in northern-central Gansu (Jinchang, Wuwei, Lanzhou, Linxia, Dingxi, Pingliang). These findings underscore the strategic value of prioritizing green infrastructure investments in identified high-response zones, particularly as enhanced urban greenery may concurrently improve living conditions to attract younger demographics.

To clarify the complex mechanisms by which variables influence aging rates in the Shaanxi-Gansu region, Table 8 offers a comprehensive summary of these relationships.

**Table 8:** Summary of the influence factors.

Factors	Variables	Impacting range		Areas impacted most strongly		Areas impacted most weakly	
		2010	2020	2010	2020	2010	2020
Demographic factors	BIR	Global	Local	Gannan Linxia Dingxi …	Zhangye Jinchang Wuwei …	Jiuquan Jiayuguan Zhangye	Xianyang Xi'an Weinan …
	MOR	Global	Local	Yulin Yan'an	Jiuquan Jiayuguan	Zhangye Jinchang Lanzhou …	Hanzhong Ankang Shangluo
	PoPP	Global	Global	Tongchuan Weinan Xi'an …	Yulin	Zhangye Jinchang Wuwei …	Linxia Gannan
Economic factor	pcGDP	Local	Regional	Yan'an	Wuwei Baiyin Lanzhou	Ankang Tianshui	Xianyang Xi'an Ankang
Medical factors	NoHI	Global	Global	Jiuquan Jiayuguan Zhangye	Zhangye Jinchang Wuwei …	Yulin Yan'an Tongchuan …	Xianyang Xi'an Weinan …
	NoCSIF	Local	Global	Tongchuan	Jiuquan Jiayuguan Jinchang …	Baoji Ankang	Xi'an Weinan Shangluo …
Environmental factors	DoGAQ	Regional	Global	Xianyang Xi'an Weinan …	Jiuquan Jiayuguan Zhangye …	Jinchang Wuwei Lanzhou …	Weinan Xi'an Shangluo …
	pcPGA	Regional	Local	Jiuquan Jiayuguan	Jiuquan Jiayuguan Baoji	Xianyang	Longnan Shangluo

This section examines how four categorical factors (demographic, economic, healthcare, and natural environmental) influence aging rates across multiple spatial scales, while deriving corresponding policy recommendations. Two notable limitations warrant further investigation. First, while out-migration represents a significant demographic phenomenon in both Shaanxi and Gansu provinces, this variable was excluded from our regression models due to data limitations. Future studies could develop systematic out-migration datasets for these regions to investigate its multi-scale relationships with population aging. Second, missing healthcare facility data were imputed using NTL data. This approach is based on the premise that NTL intensity positively correlates with the level of economic development, which subsequently associates with healthcare service provision. Although NTL datasets are often integrated with complementary data sources like point of interest (POI) records and mobile phone signaling data to enhance spatial accuracy, such multimodal integration was not feasible in this study due to data availability constraints. Nevertheless, the development of integrated methodologies leveraging multi-source datasets for healthcare infrastructure estimation remains an underexplored research frontier with substantial methodological potential.

## 5 Conclusion

This study investigated the multiscale spatial heterogeneity of population aging and its determinants, using Shaanxi-Gansu region in northwestern China as a case study. The analysis produced the following key findings.

1. **Accelerating aging**: The Shaanxi-Gansu region had already entered an aging society phase since 2010, with a significant acceleration in demographic aging by 2020. And by 2020, the proportion of the population aged 65 and above reached 13.32% in Shaanxi province and 12.58% in Gansu province, far exceeding the internationally recognized aging society threshold of 7%.
2. **Spatial–temporal heterogeneity of aging**: The Shaanxi-Gansu region exhibited distinct spatio-temporal heterogeneity in aging patterns. In 2010, lower aging rates were concentrated in northwestern (Jiuquan and Zhangye) and southwestern (Gannan Tibetan Autonomous Prefecture) Gansu, and areas in northern (Yulin) Shaanxi. By 2020, the low-aging clusters in ethnic areas of southwestern Gannan expanded, while some in northwestern Gansu (e.g., Zhangye) diminished. High-aging clusters also shifted significantly. In 2010, these clusters were predominantly located in southern Shaanxi (Hanzhong and Ankang) and parts of southern Gansu (Longnan). By 2020, new high-aging clusters emerged in Xi’an metropolitan area, while the southern clusters contracted.
3. **Spatial–temporal heterogeneity of influencing factors**: The MGWR analysis revealed distinct patterns of heterogeneity in both magnitude and scale of various factors influencing aging. Which can be summarized as follows:
  - **Demographic factors**: The effects of BIR and MOR shifted from global in 2010 to local in 2020. BIR’s influence intensified in central areas of Gansu, and shifted from negative to positive impacts in northeastern Shaanxi. MOR’s influence changed from positive to negative in areas of southern Gansu and Shaanxi as well as several areas in northeastern Shaanxi. PoPP maintained relatively stable influence intensities.
  - **Economic factor**: PcGDP transitioned from local to regional effect between 2010 and 2020, with its overall influence intensity showing a declining trend across most regions.
  - **Medical factors**: The impacts of NoCSIF transferred from a local-scale to a global-scale, while the impact of NoHI remained global-scale, and both factors shifted from (partly) positive to negative, with overall decreased influence intensity.
  - **Environmental factors**: In 2010, environmental factors exhibited regional effects, but by 2020, divergent patterns emerged. DoGAQ expanded to global-scale effects, while pcPGA transitioned to localized impacts.

The empirical findings yield four targeted policy recommendations for addressing regional aging disparities.

- **Demographic dividend maximation**: Policy interventions aimed at incentivizing childbirth may generate more significant demographic dividends in Gansu. Concurrently, implement sustained place-making strategies to enhance regional competitiveness to younger migrants through improved living conditions and employment opportunities.
- **Tiered aging expenditure**: Expenditures on aging care and services may be more allocated to central-southern Gansu, followed by northern areas of Gansu. Concurrently, eastern Gansu and Shaanxi (particularly those northern areas) require proactive fiscal preparedness to address imminent aging-induced demographic pressures.
- **Healthcare spatial optimization**: Despite quantitative improvements, local governments may need to optimize allocations of healthcare institutions and facilities through accessibility modeling.
- **Green infrastructure leverage**: Prioritize green infrastructure investments in identified high-response zones (section 4.4) to enhance urban greenery so that improved living conditions can attract younger demographics.

This geographically stratified policy framework enables the development of locally tailored strategies to address population aging and promotes sustainable regional development.

## Data Availability

Publicly available datasets were analyzed in this study. This data can be found here: https://www.stats.gov.cn/sj/pcsj/rkpc/7rp/indexch.htm; https://www.stats.gov.cn/sj/pcsj/rkpc/6rp/indexch.htm; https://eogdata.mines.edu/products/vnl/.
