# Supplementary material for: Multiscale spatial heterogeneity of population aging in relation to its influential factors: a case study in the Shaanxi-Gansu region, China
Source: Front Public Health. 2025 Mar 27;13:1551287. doi: 10.3389/fpubh.2025.1551287 (PMC11984417; doi:10.3389/fpubh.2025.1551287)
Supplement: Supplementary file 1 [file Table_1.docx]

Supplementary Material

**Supplementary Table S1** Results of multicollinearity test for variables

| Variables | VIF |
| --- | --- |
| **BIR** | 5.586 |
| **MOR** | 4.701 |
| **PoPP** | 4.732 |
| GDP | 13.557 |
| **pcGDP** | 8.873 |
| pcDIUR | 9.433 |
| NIoRR | 15.924 |
| **NoHI** | 2.860 |
| **NoCSIF** | 3.307 |
| **DoGAQ** | 4.034 |
| **pcPGA** | 3.276 |
